# Exploring factors affecting quality implementation of lymphatic filariasis mass drug administration in Bole and Central Gonja Districts in Northern Ghana

**DOI:** 10.1371/journal.pntd.0007009

**Published:** 2020-08-17

**Authors:** Alfred Kwesi Manyeh, Latifat Ibisomi, Rohit Ramaswamy, Frank Baiden, Tobias Chirwa

**Affiliations:** 1 Division of Epidemiology and Biostatistics, School of Public Health, University of the Witwatersrand, Parktown, Johannesburg, South Africa; 2 Institute of Health Research, University of Health and Allied Sciences, Ho, Volta Region, Ghana; 3 Nigerian Institute of Medical Research, Yaba, Lagos State, Nigeria; 4 Public Health Leadership Program, Gillings School of Global Public Health, University of North Carolina at Chapel Hill, Chapel Hill, North Carolina, United States of America; 5 Faculty of Infectious and Tropical Diseases, London School of Hygiene and Tropical Medicine, London, United Kingdom; Washington University School of Medicine, UNITED STATES

## Abstract

Ghana has been implementing Mass Drug Administration (MDA) since the year 2001, and Lymphatic Filariasis transmission has been interrupted in 76 out of the 98 targeted districts. The remaining districts have a microfilaria prevalence above the 1% threshold needed for the interruption of transmission. This study assesses the level of lymphatic filariasis MDA coverage and explored factors affecting the quality of implementation of the MDA in the Bole and Central Gonja Districts of Northern Ghana. A concurrent mixed methods study design approach was used to provide both a quantitative and qualitative insight. A descriptive analysis was carried out, and the results are presented in tables and charts. The transcripts of the qualitative interviews were imported into Nvivo and framework methods of analysis were used. The results were summarized based on the themes and buttressed with narratives with key quotes presented within the texts. The overall MDA coverage in Central Gonja is 89.3% while that of Bole district is 82.9%. Refusal to ingest the drug and adverse drug reactions were higher in Bole district than the Central Gonja District. The persistent transmission of lymphatic filariasis in Bole District was characterized by poor community mobilization and sensitization, nonadherence to the directly observed treatment strategy, refusal to ingest the drug due to the fear of adverse drug reactions, inadequate knowledge and misconceptions about the disease. Reported mass drug administration coverage will not necessarily result into interruption of transmission of the disease without strict compliance to the directly observed treatment strategy, strong stakeholder engagement coupled with evidence-based context-specific multi-channel community education strategies with key educational messages on the cause of the disease and adverse drug reactions. While the clock for the elimination of lymphatic filariasis by the year 2020 and meeting of the Sustainable Development Goal 3 target 3.3 by 2030 is ticking, there is an urgent need for a concerted effort to improve the fidelity of the ongoing lymphatic filariasis MDA campaigns in the Bole District of Northern Ghana.

## Author summary

After 18 years of implementing the global strategy to eliminate lymphatic filariasis in Ghana, the prevalence of the transmission of the disease is still above the threshold needed to interrupt transmission of the disease as a public health problem in some districts. This study showed that, the persistent transmission of the lymphatic filariasis in the Bole District is characterized with a refusal to ingest the drug; reported adverse drug reactions; poor knowledge and misconceptions of the disease, and poor adherence to the mass drug administration protocol. High reported mass drug administration coverage will not lead to interruption of transmission of lymphatic filariasis without strict adherence to the direct observe treatment strategy, strong stakeholder engagement coupled with evidence-based context-specific multi-channel community education strategies with key educational messages on adverse drug reactions and the cause of the disease.

## Introduction

Lymphatic Filariasis (LF) is a Neglected Tropical Disease (NTD) prevalent in poor communities and is the second leading cause of permanent disability after leprosy [1, 2]. Lf is a mosquito-transmitted parasitic disease caused by filarial nematodes; *Wuchereria bancrofti*, *Brugia malayi and Brugia timori* [3]. The microfilaria is ingested in the blood by a mosquito vector during a blood meal on the human host. Upon maturity and mating, the female worms produce millions of microfilariae that travel to the lymph and blood channels. Clinical manifestations of the disease include lymphoedema (swelling of limbs or breasts), and hydrocele (scrotal swelling) [4]. The disease undermines the social and economic welfare of affected people and their communities.

In the year 2000, the World Health Organization (WHO) launched the Global Program to Eliminate Lymphatic Filariasis (GPELF) with the goal to eliminate LF as a public health problem in all disease-endemic areas by 2020. The primary GPELF strategy recommended was based on mass drug administration (MDA) of antifilarial medications that reduce microfilaraemia rates in endemic areas to levels below those needed for sustained transmission by mosquitoes.

The key strategy employed in sub-Saharan Africa is annual mass treatment with single-dose diethylcarbamazine (DEC) or ivermectin (IVM) in combination with albendazole (ALB) for 4 to 6 years with effective population treatment coverage of ≥65% [2, 5, 6]. Successful implementation of LF MDA has been reported in some parts of the world [7, 8]. The implementation of MDA in Ghana started in 2001 at the village/community level using community drug distributors. The MDA is a community-directed treatment approach where the drug is delivered to individuals in their homes [9].

The WHO recommended a systematic approach to interrupt transmission of LF. The strategybegins with mapping the distribution of LF to identify areas in need of MDA, followed by five or more years of MDA, a period of post-MDA surveillance, and verification of LF elimination [2].

The post-MDA coverage surveys are conducted at the population level by LF elimination programs to validate reported MDA coverages rates, and ascertain reasons for non-compliance. The post-MDA survey also identifies challenges with the drug supply chain and distribution systems, and assess the effectiveness of education, information and communication strategies to improve subsequent MDAs [2].

After completing five rounds of MDA, a pre-transmission assessment survey is conducted to measure the effectiveness of the MDAs and to identify if the endemic district will qualify for transmission assessment survey (TAS) which is conducted after six effective MDA rounds. The TAS are conducted according to the WHO recommended guidelines to determine when infections have been reduced below the targeted thresholds [2].

After about 15 years of implementing the MDA program in Ghana, a cumulative total of 74 million people in 98 endemic districts have been treated. Of the 98 endemic districts, a total of 76 districts achieved interruption of transmission of the disease with a microfilaraemia prevalence rate of <1%, and passed a transmission assessment survey and established post-MDA surveillance. The MDA was stopped in those areas by April 2016. The interruption has been attributed to high levels of MDA coverage in these districts. The remaining 22 districts that have prevalence above the 1% LF threshold needed to interrupt transmission of the disease after 15 years of MDAs and are now referred to as LF "hotspot" districts [10, 11]. Factors contributing to thepersistent transmission of the disease given the efficacy of the drug coupled with high reported MDA coverage in the remaining districts are unknown.

The level of adherence to the MDA process is a key determinant of the success of the MDA program [7, 12, 13]. Factors such as systematic refusal to ingest the drug and nonadherence to the MDA protocols present a, programmatic severe obstacle for the success of the LF program [7, 8, 11, 14, 15]. In some previous studies, knowledge about LF was found to be positively associated with ingestion of the LF drug in some studies [14, 16]. Other studies have shown that factors that contribute to MDA non-compliance include: the poor knowledge about the disease and the MDA among the endemic population, fear of adverse effects from treatment, distrust of government programs, a general dislike of taking drugs, low motivation of drug distributors, lack of knowledge of the disease, and inadequate communication on the rationale of MDA [17–19].

While the WHO considers at least 65% coverage to be an effective MDA round, researchers have suggested that, for the success of the global MDA strategy to eliminate the disease, there is the need for key players to comply with the ‴ 'program's guidelines and to achieve and sustain high levels of coverage (>80%) as shown in Haiti [8]. In Ghana, a host of issues affecting the MDA program have been identified and need to be individually explored and addressed to improve the MDA process. Some of these issues include community availability and participation in the MDA, community/stakeholder engagement before and during the MDA[11, 20, 21], other health system challenges such as poor supervision due to heavy workload, delays in releasing funds, and logistics and minimal monetary incentive to drug distributors [20–23].

While the clock is ticking for the Sustainable Development Goals (SDGs) 3 target 3.3 (which aims to end the epidemics of AIDS, tuberculosis, malaria and neglected tropical diseases and combat hepatitis, water-borne diseases and other communicable diseases by 2030) [24], there is a need for a concerted effort and focused attention on how best to administer the drugs to the people living in LF endemic communities. Hence, this study assessed the level of MDA coverage; explored factors that contributed to the interruption of the transmission of LF in Central Gonja District; and the persistent transmission of LF in the Bole District of Northern Ghana. The aim was to help design and implement a quality improvement strategy for more effective MDA by way of learning from a district that interrupted the transmission of LF, as well as to understand factors contributing to the persistent transmission of LF in a hotspot district. Quality MDA implementation, as used in this study, is defined as implementing the LF MDA according to the WHO recommended guideline, taking into consideration context-specific factors.

## Methods

### Ethics Statement

The study was reviewed and approved by the Human Research Ethics Committee (medical) of the University of the Witwatersrand, Johannesburg, South Africa (Clearance Certificate No. M170219) and the Ghana Health Service Ethics Review Committee (Clearance Certificate No. GHS-ERC: 04/02/2017). Permission was obtained from the Neglected Tropical Diseases (NTDs) program of the Ghana Health Services and the District Health Administration of both the Bole and Central Gonja Districts for the extraction and use of the secondary LF data. All study participants were adults, and informed consent was provided to all participants in the study. All participants also consented to ehave de-identified quotes used in a publication in the consent form.

### Study site

**Fig 1:**
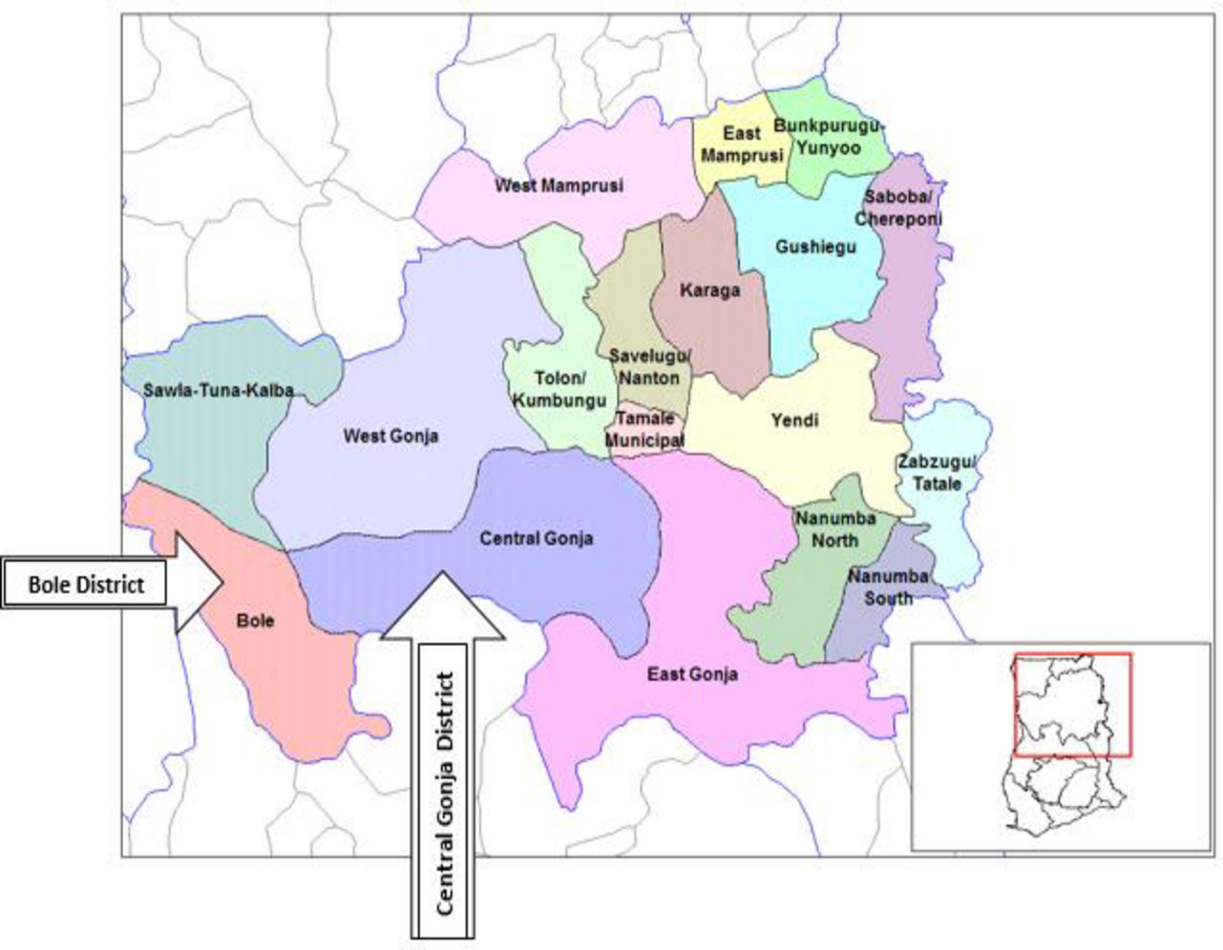
Location of Bole and Central Gonja districts in Northern Ghana.

The study was conducted in the Bole and Central Gonja districts of Northern Ghana as shown in Fig 1. These two districts were purposively selected due to the persistence and interruption of the disease transmission in the two districts, respectively.

The Bole district is one of the 22 districts in Ghana where the transmission of the LF persisted after more than 15 years of MDA. A district hospital is situated in Bole (the district capital). Health services are divided into six sub-districts with each sub-district served by a health centre. Also, the Catholic Church runs a primary health care program in the Bole township. There are also six operational Community-based Health Planning and Service (CHPS) compounds within the district [25]. The Bole district also has a community health ‴nurses' training school. The district has a baseline microfilaraemia prevalence of 0.3%. The current microfilaraemia prevalence is 1.9%, but some communities have a prevalence rate as high as 5.9% (source: Neglected Tropical Diseases Program, Accra, Ghana).

The Central Gonja district, on the other hand, is among the 76 districts in Ghana that interrupted the transmission of LF. The district is served by a District Hospital supported by health centres located in each of the five sub-districts. The Tamale Teaching Hospital serves as a referral centre for medical conditions. The district also has a Health Assistance Training School, which trains professionals for the health sector [26]. These health facilities provide both preventive and curative health services to the population. While the baseline microfilaraemia prevalence in Central Gonja was 3.7%, the current prevalence is 0% after testing 592 individuals in the district (source: Neglected Tropical Diseases Program, Accra, Ghana).

### Study design

A concurrent mixed methods study design approach was used to provide both a quantitative and qualitative insight. This involves a separate collection and analysis of quantitative and qualitative data.

### Quantitative method

The quantitative arm of the study is made up of secondary data of 70,591 and 106,266 residents from Bole and Central Gonja Districts of Northern Ghana, respectively who took part in the 2016 and 2014 MDAs. A primary survey data collected from 446 randomly selected community members in both study districts in 2017.

The process of selecting the participant is described elsewhere [27]. The primary data includes information on the socio-demographic characteristics of participants, knowledge of how LF is acquired, knowledge of signs associated with LF, knowledge of LF prevention, and misconceptions about LF.

These variables were measured by correct answers to signs associated with LF, LF prevention methods, and how LF is acquired (as shown in the questionnaire in S1 Appendix).

Comprehensive knowledge of LF was estimated by the ability to identify correctly at least two significant ways of preventing LF transmission, mode of LF transmission, and rejecting at least two misconceptions about how LF is acquired from section B of the questionnaire in the S1 Appendix.

For this study, coverage refers to the percentage of targeted persons who receive the MDA tablets, and compliance denotes the percentage of a targeted population who ingested the medications.

A descriptive analysis was carried out in Stata version 14.2 and Quantum Geographic Information System (QGIS) [28].

### Qualitative method

Twelve (12) out of 96 communities from three sub-districts which had MDA coverage below 80% were purposively selected in the Bole District. Four (4) out of 84 communities from four sub-districts with MDA coverage above 80% were also purposively selected from the Central Gonja District to explore factors that contributed to the interruption of transmission of the disease. This strategy was employed to gain a deeper understanding of the relevant factors affecting the quality of implementation of the LF MDA strategy.

Twenty (20) in-depth interviews (IDIs) were conducted with health workers made up of District Directors of Health Service (DDHS), Sub-district Heads of Health Service (SHHS), and Disease Control Officers (DCOs).

The IDIs were also conducted with Community Drug Distributors (CDDs) to understand the perspective of health and frontline workers regarding factors affecting the quality of implementation of MDA in the study area. Besides, 16 key informant interviews (KIIs) were conducted with opinion leaders and non-compliance community members in both districts, as shown in Table 1. The CDDs are community selected individuals who were trained on enumeration of households, LF treatment eligibility, exclusion criteria for treatment, drug storage and safety, referral process of severe adverse drug reactions, and record keeping.

**Table 1:** Participants and type of interview.

Respondent Type	Central Gonja District		Bole District	
	IDI*	KII**	IDI	KII
District Directors of Health Service (DDHS)	1	-	1	-
Sub-district Heads of Health Service (SHHS)	3	-	2	-
Disease Control Officers (DCOs)	2	-	2	-
Community Drug Distributors (CDDs)	5	-	4	-
Noncompliance Community Members	-	5	-	3
Chiefs/Opinion Leaders	-	4	-	4
**Total**	**11**	**9**	**9**	**7**

* IDI = In-depth Interview

** KII = Key Informant Interview

Semi-structured interview guides were used for the data collection. They were translated from English to the local language before the fieldwork began. Back-to-back translation strategy was used to ensure that the versions in English were the same as those in the local languages. The guides covered broad areas of knowledge of causes and transmission of LF, perception of LF drug, knowledge and awareness of MDA, implementation process of MDA, community understanding; and perception of MDA.

The interviews were conducted in venues chosen by participants. This was generally in their offices and homes. The interviews took between 60 and 90 minutes. All interviews were recorded using digital voice recorders, translated and transcribed verbatim. Permission was sought from the research participants for their participation and to record the IDIs and KIIs using digital voice recorders. Field notes were also taken, which were turned into data documents for analysis.

The IDIs and KIIs with CDDs, Chiefs/Opinion leaders and non-compliance community members were conducted in the local language and translated into English by two independent language experts during transcription. The corresponding transcripts were compared and reviewed by independent language experts.

All field notes and the transcriptions were made anonymous by removing all identifying information, and each participant was assigned a unique identifier. All the qualitative narrative data were then inputted into a Microsoft Word processor and exported into NVivo Version 10 for Windows for coding. Framework analysis was employed to analyse the data [29, 30]. The results were summarised based on themes that emerged from the data with key quotes from participants as presented within the text.

## Results

### Mass drug administration coverage

**Fig 2:**
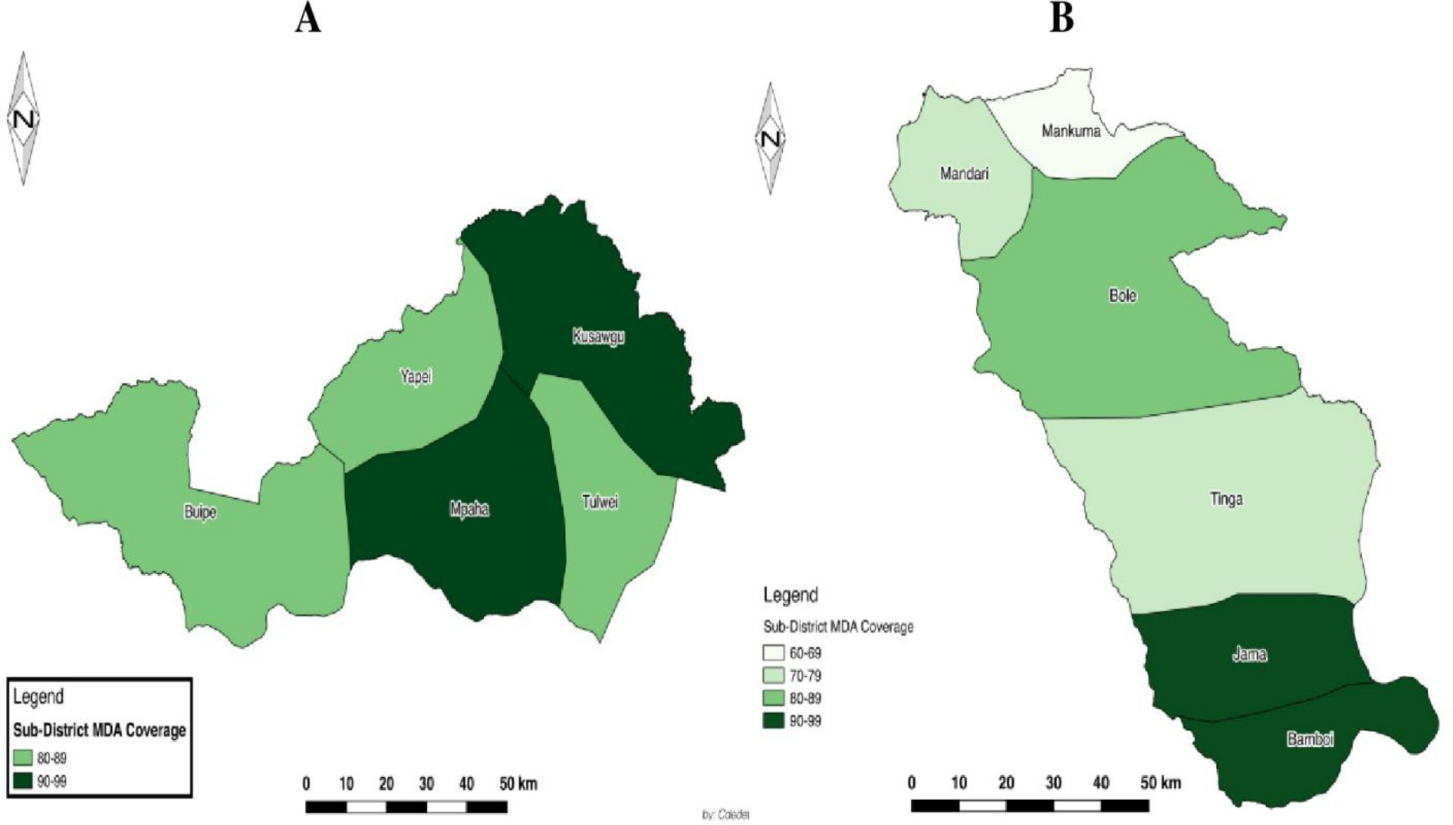
Patterns of Sub-district Mass Drug Administration Coverage in Central Gonja (A) and Bole (B) Districts.

As shown in Fig 2, all sub-districts in Central Gonja had an MDA coverage of above 79%, while some sub-districts in Bole District recorded MDA coverages below 70%. More specifically, in Bole District, while Bamboi and Jama sub-districts had coverages of above 95%, while Mandari, Tinga, and Mankuma had coverages below 80%. Although the overall MDA coverage in Bole District is high (82.0%), there were some communities with coverages as low as 47.3% (see S1 Table).

### Socio-demographic characteristics of survey participants

Except for educational level and occupation there are no significant differences in the socio-demographic characteristics of the study participants in the two districts, as shown in Table 2. More than half (50.5%) of the survey participants from the Central Gonja District were female. The mean age of the participants from Central Gonja is 40.5 years (13.5 standard deviations). Whereas in Bole District, there were 52.3% female participants with an average age of 43.9 years (25–64 range) as shown in Table 2.

**Table 2:** Socio-demographic information of survey participants.

	Central Gonja District		Bole District		P-Value
	(n = 224)		(n = 222)		
Variables	Frequency	Percentage	Frequency	Percentage	
**Sex**					0.703
Male	111	49.6	106	47.8	
Female	113	50.5	116	52.3	
Mean Age (Range)	40.5 (28–59)		43.9 (25–64)		
**Marital status**					0.692
Married	178	79.5	173	77.9	
Not married	46	20.5	49	22.1	
**Occupation**					0.008*
Farmer	170	75.9	158	71.2	
Trader	32	14.3	27	12.2	
Public servant	11	4.9	5	2.3	
Unemployed	6	2.7	15	6.7	
Artisans	5	2.2	17	7.7	
**Religion**					0.631
Christian	89	39.7	98	44.1	
Moslem	110	49.1	100	45.1	
Traditional	25	11.2	24	10.8	
**Level of education **					<0.001
No Education	110	49.1	158	71.17	
Primary	90	40.2	23	10.4	
Junior High Sch.	9	4.0	27	12.2	
Senior High Sch. and tertiary	15	6.7	14	6.3	

* Fisher's exact test

### Knowledge on lymphatic filariasis

The survey result presented in Table 3 shows that there is a significant difference in knowledge about LF among the study participants in the two study districts. The comprehensive knowledge of LF among the participants in Bole District is higher compared to that of Bole District.

**Table 3:** Knowledge about lymphatic filariasis in Central Gonja and Bole Districts.

	Central Gonja District (n = 224)		Bole District (n = 222)		
	Frequency	Percentage	Frequency	Percentage	P-Value
**Have correct knowledge of how LF is acquired**					<0.001
Yes	200	89.2	75	33.9	
No	24	10.8	147	66.1	
**Have correct knowledge of signs associated with LF**					<0.001
Yes	217	96.9	127	57.1	
No	7	3.2	95	42.9	
**Have correct knowledge of LF prevention**					<0.001
Yes	146	65.3	82	37.1	
No	78	34.7	140	63.0	
**Misconception about how LF is acquired**	** **	** **	** **	** **	<0.001
Yes	35	15.5	131	59.2	
No	189	84.5	91	40.8	
**Comprehensive knowledge of LF**					<0.001
Yes	178	79.7	70	31.5	
No	46	20.3	152	68.5	

The quantitative is similar to the qualitative results where some of the Community Drug Distributors (CDDs) who participated in the IDIs in Bole District were not knowledgeable about the causes of LF. Also, none of the participants in the non-compliance IDIs knew the causes of LF due to a lot of misconceptions about how the disease is contracted in their communities. Some respondents were superstitious about LF by attributing it to curses for stealing and doing something evil against someone. The following comments indicate this:

*"It is a result of the curse*… *so if a person steals something*, *they are cursed with the disease"*. (Noncompliant, Bole District).

*"There is a hole in this community when you step in it*, *and you get LF"* (Opinion leader, Bole District).

Some of the CDDs who participated in the IDI in Bole District were not knowledgeable about the specific cause of LF as shown in the quote:

*"I 'don't know anything about it; I only see the leg swell big and when I ask they say is kiapeni' (LF) and I 'didn't ask what causes it"* (CDD, Bole District).

### Prevention and treatment measures

The respondents indicated that the disease could be prevented by sleeping under treated bed nets, keeping clean environments, going for regular check-ups, and taking the LF drug amongst other measures.

Although some respondents thought the disease could be prevented by eating hot food, others were also of the view that taking the drugs would automatically prevent them from getting the disease. These were the comments following the question about prevention:

*"By taking the LF drugs*, *we have been receiving*… *So those that refuse to take the drug 'wouldn't be able to prevent the disease*, *but we those that take the drug automatically prevents our system from getting LF"* (CDD, Bole District).

*"The disease can be prevented by sleeping under a mosquito net*, *intake of LF drugs*, *and regular check-up in the hospital can all prevent LF infections"* (CDD, Central Gonja District).

### Perceptions of LF on livelihood and stigma

The respondents perceived LF as a horrible and dangerous disease. Hence they found it challenging to buy food from people who have clinical manifestations of the disease and sell food. This results in affected people becoming jobless. It is also clear from the comments that people affected by the disease live in isolation and tend to be lonely. No one wants to associate with them, and they are mostly abandoned by their communities as shown in the key quotes:

*"They see it as a dirty disease*… *if someone is suffering from it and sells food they 'won't buy the food*… *people with LF stay away from people*… *they 'don't want to associate themselves with people"* (Noncompliant, Bole District).

*"The disease makes life very difficult for people*… *they 'can't work or even do anything again*… *and your friends will not come close to you again*.*"* (Opinion Leader, Central Gonja).

*"It is a very bad thing*… *the people who have it are always lonely and isolated*.*"* (Health worker, Bole District).

### Reasons why this disease still exists

Health workers, the noncompliant and CDDs stated that why the disease still exists in the communities was because community members refused to take the LF medication. This illustrated by the following quotes:

*"I think those suffering from the disease 'don't take the drug that is why*… *if they were to be taking the drugs by now they would have been treated"* (Noncompliant, Bole District).

*"Well*, *a lot of people believe that the disease can be acquired when a healthy person comes into contact with someone with the disease so they 'don't like taking the drug"* (Health worker, Bole District).

*"A lot of people in the community give excuses when we are distributing the drug*… *they 'don't want to take the drug*… *I think that is why people still have the disease"* (CDD, Bole District).

### Knowledge about the mass drug administration exercise

The results in Table 4 show that there is a significant difference in the level of knowledge about the mass drug administration exercise among the study participants from the two study districts. Participants from Central Gonja District showed a high level of knowledge and participation in the MDA exercise compared to those from Bole District. While adherence to the direct observation treatment (DOT) strategy is significantly higher in Central Gonja District than in Bole Districts, the fear of side effects of the drug as the reason for the Refusal to ingest the medicine was higher in Bole District compared to Central Gonja District.

**Table 4:** Knowledge of lymphatic filariasis mass drug administration in Central Gonja and Bole Districts.

	Central Gonja District (n = 224)		Bole District (n = 222)		P-value
Variables	Frequency	Percentage	Frequency	Percentage	
**Awareness of LF MDA**					<0.001
Yes	173	77.2	102	46.0	
No	51	22.8	120	54.1	
**Source of MDA information**					<0.001*
Radio	2	1.3	2	2.3	
Health workers	58	33.5	6	5.4	
Posters	12	6.7	0	0.00	
Family members	2	0.9	7	6.7	
Church/mosque	23	13.4	18	18.0	
Community volunteers	39	22.3	37	36.5	
Gong-gong	35	20.5	14	13.5	
Neighbors/friends	2	1.3	18	17.6	
**Was there any public education before MDA**					<0.001*
Yes	154	89.0	40	39.6	
No	18	10.4	51	50.0	
Don’t know	1	0.6	11	10.4	
**Have you ever taken the LF drug**					<0.001*
Yes	210	93.8	98	44.1	
No	10	4.5	82	36.9	
Can't remember	4	1.8	42	18.9	
**How was the drug administered**					<0.001*
Direct observation treatment	221	98.7	140	63.1	
Given to beneficiary to take at his/her convenience	3	1.3	82	36.9	
**Why some community 'members' Refusal to ingest drug**					<0.001*
Fear of side effects	140	62.5	143	64.4	
Level of knowledge of the disease	10	4.5	20	9.0	
'Don't think they will get the disease	9	4.0	17	7.7	
Think only sick people should take the drugs	6	2.7	15	6.8	
Too many drugs	16	7.1	18	8.1	
Religious beliefs/superstition that oppose medication	3	1.3	6	2.7	
Taking other medication	19	8.5	2	0.9	
Have taken the drugs far too many times	21	9.4	1	0.5	

* Fisher's exact test

In the qualitative study, participants generally exhibited good knowledge of the MDA exercise. In the IDIs, the non-compliance community members and health workers knew the purpose of the MDA exercise by indicating that the exercise was carried out to prevent LF. Both health workers and the CDDs interviewed were able to outline the time the program commenced. According to them, the MDA is a yearly program that is undertaken between April and July (the beginning of the wet season). However, they say this is not favourable because this coincides with the planting season, which then becomes a major challenge in terms of availability of community members carry out the program. This is expressed in the quotes:

*"The last distribution that we did occurs during the rainy season which is not the best as most people go to the farm"* (CDD, Bole District).

*"The exercise is done at the beginning of the farming season*… *the kind of work (farming) the people do here is a challenge as most of them sleep at their farms during the farming season"* (Health worker, Bole District).

According to the respondents, before the MDA exercise, certain activities need to take place. Amongst them are cascade training, announcements to community members about the day of the exercise and registering various household members as reported in the following comments:

*"Before the distribution of the drugs*, *I make an announcement at the Mosque*, *and at information centres to inform the people about the date the medicine will be distributed"* (CDD, Bole District).

*"Before the MDA exercise we were trained at Bole and we also trained the volunteers here in Tinga*. *The drugs and other materials were sent to us after the training"* (CDD, Bole District).

According to respondents, the training took the form of illustrations and presentations. They were shown photos of LF, the drugs were introduced, and they were taught how to distribute them during the exercise. They were also taught how to measure the height of people and how to administer the drugs, and also to the register pregnant women, all births and deaths and the recording of possible causes of death.

IDIs with drug CDDs in Bole District showed that announcements were made by the CDDs in mosques, churches, information centres and by using the 'gong 'gong' (a traditional means of giving information to the community) before the drug distribution. This is explained by the following quote:

*"Most often*, *it is the hospital that informs me as a volunteer and I will also let them make announcements through gong-gong beating*. *Also*, *it is me the volunteer who will move from house-to-house to inform the people again about the drug and its purpose before they will accept and take the drugs"* (CDD, Bole District).

The qualitative results show that the announcements before the MDA only informed the community about the date of the drug distribution but did not focus on: educating the community about the importance of the MDA, elimination of LF, adverse drug reaction and management, the importance of DOT, and why all eligible community members must take part.

Community engagement and involvement in the planning of the MDA was also not mentioned in any of the responses.

In Bole District, announcements about the MDA programs were made by CDDs, whereas in the Central Gonja District, the announcements and education were done by the health workers as shown in the following quotes:

*"Before the distribution of the drugs*, *the CDDs make an announcement at the Mosque*, *and information centres to inform the people about the date the medicine will be distributed"* (Health worker, Bole District).

*"We first go for training at the district level then we come to train at the sub-district level*…*then to the community to give health information to community members before the actual day of distribution"* (Health worker, Central Gonja District).

### Activities during the MDA

Activities during the MDA exercise include: identification of homes, counting of individuals, height measurements were taken, and establishing other inclusion criteria (pregnant women, seriously sick people and under-height children were excluded from taking the drug).

With the drug administration process, respondents pointed out that people access the drug from door to door distribution and not at a central point. Thus, the CDDs move from house to house to deliver the drugs to the community members. The following comments were reported:

*"I start by planning my movement and how to identify homes that have not been completely administered and those that are completed*. *I do this by numbering the various homes before entering and circle it if completely administered*, *but I leave it open if I have some household members missing or absent and I have to return to that house to attend to them*. *I also decide on what time is best and appropriate to get the people at home to reduce the amount of movement to and fro for absent people*. *After taking their heights and giving them the drug*. *I also get myself some indelible ink which I put on a finger of those who have taken the drug to prevent overdose"* (CDD, Bole District).

*"We do not give the drug to children who are under-height*, *pregnant women and seriously sick persons*… *but aside these groups of persons*, *every other person takes it"* (CDD, Bole District).

### Adherence to the directly observed treatment strategy

Some of the CDDs interviewed in Bole District interviewed leave the drug for absent household members and people who insist on ingesting the medicine at their convenience. On the other hand, CDDs in Central Gonja District adhered to the DOT strategy by ensuring that all eligible household members ingest the medicine in their presence as indicated by the following:

*"Most of them collect the drug from you telling you that they will take it at their convenience*, *but it was later revealed that most of these people do not take the drug after all"* (CDD, Bole District).

*"Since everybody in the community needs the drug and I know most of the people if someone is not at home at the time of the distribution I normally leave his own[drug] with someone in the house for him"* (CDD, Bole District).

*"I explain the purpose of the MDA to every household member and I make sure every eligible household member swallows the drug with water in front of me before I leave the house"* (CDD, Central Gonja District).

### Perceptions and side effects of the drug

According to the CDDs, community members have the belief that the drug has some side effects. Some of the adverse drug reactions reported include vomiting, dizziness, swollen testicles and body itches as reported:

*"People complained about swollen testicles and some people say when they take it [the drug] they have some itches in their body [*…*] some people also say they have bodily pain when they ingest the drug"* (CDD, Bole District).

*"They say they were vomiting*, *and feeling dizzy*, *but sometimes most of them 'don't eat before taking the drug*. *But we have not recorded any serious side effects that should scare somebody enough not to take the drug"* (Health worker, Bole District).

### Challenges faced by CDDs and health workers

The challenges encountered by the health workers and drug distributors were lack of transport to cover the communities, frequent breakdowns and sometimes no fuel for motorbikes They also struggled with bad road networks due to potholes and flooding and a lack of motivation and incentives. Some CDDs said they were attacked by community members who experienced some form of reaction to the drug. This is revealed in the following comments:

*"We 'don't have motorbikes; fuel is number two*… *flooding of roads to some communities is also a big problem here"* (CDD, Bole District).

*"They usually complain and sometimes attack me*… *because of the reactions or side effects they have after taking the drug*… *I try to explain or convince them but at times it 'doesn't work"* (CDD, Bole District).

### Reasons for refusals

The qualitative result shows the refusals of the community members refused to ingest the drugs due to misconceptions of the drug were, such as the drug was a method for family planning, the side effects of the drug, the perception that it kills people, and others superstitions and traditional inclinations.

It is worth noting that the involvement of opinion leaders in the MDA process that helped people to refrain ingesting the drug in Central Gonja District and presented in the quotes:

*"Some people say it is a disguised family planning drug"* (Health worker, Bole District).

*"The last time I took the medicine I had lots of side effects … I was vomiting*, *itching and had rashes on my body … so I stopped taking it*. *Due to this problem and I know many people who do not want to take it due to the same problem"* (Noncompliant, Bole District).

*"Yes*, *at times*, *some people refuse to take the drug for fear of side effects and some other reasons but when I bring in the assemblyman and we explain to them there agree to participate"* (CDD, Central Gonja District).

### Suggestions on how to reach community members and improve MDA

The respondents suggested that to reach more people, education and awareness creation about the MDA should be intensified. Also, there should be adequate transport, increased motivation, the involvement of community leaders and a strategy to involve males who drink alcohol needs to be devised. This is shown in the following responses:

*"I think*, *there is the need to intensify sensitization to educate the people more about the safety and possible side effects of the drugs*… *the community need to understand that the side effects are normal and will not harm them … they should also be made to understand the importance of taking the drugs"* (Health worker, Bole District).

*"The involvement of the community leaders during the MDA is very important*. *Their involvement makes our work very easy"* (CDD, Central Gonja District).

According to the respondents, the MDA can also be improved through education and sensitization on the safety of the drug and side effects to allay the fears of community members and the involvement of community leaders. Some respondents also indicate the need to reduce the quantity of the drugs and use bed nets to be used to lure community members to take the drugs. This is indicated in the quotes:

*"They should distribute bed nets when they are coming to distribute the drugs and it will encourage people to take the drugs*… *people in this community find it difficult to believe issues so if they could find time to have meetings and educate them it will help"* (Noncompliant, Bole District).

*"More education should be done before the distribution so that people can take it [the drug] without any fear"* (CDD, Bole District).

*"They should reduce the number of drugs they give; they give plenty and ask you to take all"* (Noncompliant, Bole District).

## Discussion

### Mass drug administration coverage

The two study districts recorded MDA coverage above the recommended 65% coverage needed for interruption of transmission of the disease. This high MDA coverage reported in the current study is consistent with the findings by other Ghanaian studies which reported MDA coverage rates above 65% [31, 32].

High MDA coverage, which does not translate into interruption of transmission of the disease after more than 4–6 rounds of MDA, was found to be associated with recording errors as reported by other researchers [21]. It has also been shown that for programs where the DOT strategy is not strictly followed, distributed drugs may not necessarily be consumed [33–35]. Due to the evidence of nonadherence to the DOT strategy by some CDDs in Bole District, the reported MDA coverage may not reflect accurate MDA coverage in the district. In this situation, all distributed drug may not necessarily be ingested. This could explain why the reported MDA coverage is high (above 65%) in Bole District but the transmission of the disease persists. The reported coverage in this situation will reflect distributed drugs as opposed to ingested drugs because the DOT strategy was not strictly being adhered to by CCDs. This nonadherence could also be contributing to maintaining a reservoir of LF infection in Bole District hence the persistent transmission of the disease in the area.

The MDA coverage for all sub-districts and communities in the Central Gonja District was very high compared to communities and sub-districts in the Bole District. This could also be another explanation for the persistent transmission of the disease after many rounds of MDA in the Bole District. This is consistent with existing evidence that low treatment-coverage rates in MDAs place the success of elimination programs at risk [3] and have been linked to failure in drug distribution, lack of perceived treatment benefit by the endemic population and fear of adverse drug reaction [17, 36–38]. This finding is also in line with the GPELF, which advocates for the treatment of entire endemic communities to achieve its elimination targets [5]. The low MDA coverage in the Bole District compared to Central Gonja District could also be due to refusal to ingest the drug, as confirmed by the qualitative results hence the persistent transmission of disease in Bole District.

### Refusals and adverse drug reaction

The refusal of people to participate in Bole District to ingest the medicine was explained in the qualitative study as misconceptions about the cause and treatment of the disease, fear of adverse drug reaction, inadequate knowledge about the disease, and religious beliefs.

The refusal to ingest the drug by eligible people in Bole District as compared to Central Gonja could also be a contributing factor to the low coverage in some sub-districts and communities as such continuous refusal to ingest the drug could lead to a perpetual transmission of the disease. Previous research in India has associated the continuous transmission of LF with systematic non-compliance and refusal to ingest the drug [39]. The non-compliance has facilitated continuing infection of microfilariae in certain communities in Haiti and Nepal [15, 40, 41]. This probably explains why the disease is still being transmitted in the Bole District after more than six years of MDA.

Previous researchers also showed that when communities did not receive correct and adequate information about adverse drug reactions, the consequences were detrimental to the success of the MDA [7]. Studies from India have also demonstrated that insufficient information about the MDA [6, 42] and rumours about adverse drug reactions, when not addressed, persisted and negatively affected the MDA in neighbouring areas as well [43, 44]. It has,been suggested that side effects of MDA must be communicated and reiterated at the time of the pre-MDA mobilization and during the drug distribution [7].

The occurrence of adverse drug reactions and its rumours in Bole likely contributed to the low MDA coverages as it could make people hesitant to ingest the drug as shown in Sri Lanka, India, The Philippines, Papua New Guineas and Indonesia [12, 13, 18, 36, 37, 45–53]. The fear of adverse drug reaction in Bole is also an indication of inadequate information about MDA treatment and failure to understand that such severities are rare in LF MDA. It has also been asserted that the failure to convey information about adverse drug reaction to community members and families negatively affects coverage and acceptance of MDA [7].

However, it should be emphasized that the self-reported adverse drug reaction in the Bole District could also be an indication of the high levels of transmission of the disease and parasites. In other words, people with higher levels of the filarial parasite tend to have adverse drug reactions indicative of the drugs killing their circulating parasites [11, 54]. In effect, the degrees and severity of adverse events following ingestion of the LF drug increases with increasing microfilarial loads and increased microfilaricidal efficacy [55]. Conspicuously, therefore, this adverse drug reaction in Bole District could also confirm the high levels of transmission of the disease.

### Non-eligibility and absenteeism

In this study, non-eligible participants in the MDA were under-height, pregnant or lactating mothers which factored higher in Bole District compared to Central Gonja District. Notably, a community-based study associated continuing transmission of LF in Haiti with high LF infection rates among young children, most of whom were under-height and did not meet the inclusion criteria for the ' 'district's MDA [40]. In addition, although not WHO criteria, all breastfeeding mothers were considered ineligible for treatment. As shown elsewhere (Egypt, Haiti, Indonesia) this exclusion of lactating mothers from the MDA might have contributed to the large numbers of ineligible persons who can maintain a reservoir for the persistent transmission of the disease as shown elsewhere [33–35].

Again, the result shows that in both Ghanaian districts of this study, there were people who met the eligibility criteria but who did not take the drug due to being absent at the time of the drug distribution. One explanation given in the qualitative study is that the drug is normally distributed at the beginning of the busy farming season when most community members are absent at the time of drug distribution. This finding confirms earlier studies which suggest that the time allocated for MDAs and its mopping-up activities was not sufficient to cater for people who were temporarily absent at the time of drug distribution [46, 56, 57]. It has also been shown in other studies that insufficient time adversely affects MDA coverage [6, 45, 46, 58]. The short time allocated for the MDA exercise makes it difficult for the drug to get to all eligible recipients in Bole District.

### Knowledge of mass drug administration activities

The result revealed a significant difference in knowledge of mass drug administration activities among participants from the two study districts.

The health workers and the CDDs were able to outline the activities carried out before and during the MDA. They describe cascaded training for health workers and CDDs, community announcements, updating of LF registers and distribution of the drugs. However instead of the community sensitization and education process prescribed by the MDA strategy, often only the date of commencement of the MDA is announced to the communities and, in some instances, it is not done by health workers. This no doubt creates a knowledge gap about the disease and would account for the prevailing attitude and perceptions of the MDA program in the Bole District. This finding is consistent with the results of studies conducted in Kenya, Ghana, India, Indonesia and Haiti [7, 8, 21, 35, 59–61]. Therefore, community sensitization and mobilization through evidence-based, multi-channel communication approaches with key messages on treatment and side effects combined with high visibility of the MDA activities is necessary for more effective MDA and elimination of LF in Bole District.

A review of factors influencing compliance of MDA for the elimination of LF by researchers found that ingestion of the pills is positively associated with advanced knowledge of MDA [7]. Hence, information about LF, the pills, and their distribution needs to be frequently available before the commencement of the MDA.

A study in Nepal showed that awareness of MDA campaigns had significantly increased the level of compliance among participants and that respondents who had knowledge of side effects during MDA campaigns had a lower prevalence of non-compliance [41]. Similarly, the current study shows that education and information on the importance of the program and the potential drug reactions were not properly communicated to the community members in Bole District. Lack of education in the study area led to physical and verbal attacks on CDDs by individuals who had adverse drug reactions. These findings are in line with the WHO recommendation that community involvement in health programs will not only help to remove the burden of dependency that characterizes the health development work but also creates general awareness among local people about the need for their involvement in all forms of health development [62].

There was no mention of community engagement and involvement among the MDA activities listed by the health workers and the CDDs from Bole District. Yet results from Central Gonja District and evidence elsewhere shows that the involvement of local leaders, national institutions, religious and non-governmental organizations and political authorities in the MDA activities enhances the participation, coverage and compliance of the MDA [7, 8, 59, 63]. It is likely, therefore, that non-involvement of some of these key stakeholders contributed to non-compliance and challenges encountered by volunteers during the program.

The current study also revealed misconceptions amongst the noncomplying members of communities in Bole District. They are of the view that the drugs are supposed to be taken by people who have a chronic illness or clinical manifestations of the disease. The delusion about LF is an indication of poor education about the disease and the near-silent treatment of the MDA activities in the Bole District.

The uptake of the MDA programs in Bole District is being jeopardized by lack of health education. Bridging the knowledge gap with appropriate educational intervention is cardinal to changing perceptions and popularly held beliefs about the in Bole District. Therefore the provision of clear information on potential drug reaction and its management in the LF endemic communities is crucial in restoring confidence in the MDA intervention in the district.

### Knowledge of lymphatic filariasis

In this study, it is revealing to know that, all LF MDA noncompliant individuals interviewed did not know the exact cause of the LF disease. Some of the trained drug distributors were unable to indicate the exact cause of the disease which indicates the level of training in the area, the poor community sensitization and social mobilization, as well as inadequate information, education and communication (IEC) strategies. Studies had shown that social mobilization and awareness campaigns to inform communities on the process before and during community-based interventions contributed to the success of such programs [8, 59]. Drug distributors at the interface, therefore, are critical to the ' 'program's success with the targeted population through adequate training and appropriate knowledge of the LF disease [7, 8]. Inadequate training of CDDs and health workers results in nonadherence to the DOT strategy where pills were left in houses without adequate instructions for family members who were not present at the time of drug distribution [18, 43, 58].

### Effect of misconception

Owing to misconceptions about the disease, social stigmatization and self-imposed restrictions associated with the disease in the Bole District, unemployed individuals are living with the disease as victims of the disease. They are considered invalids which other community members refuse to support by buying their goods or using their services. There are also perceptions and strongly held beliefs that people with the diseases have done committed crimes, and therefore nobody wants to associate with them since they are cursed. These misconceptions and firmly held beliefs about the disease are other indications of the inadequate distribution of information about the disease after many years of program implementation in Bole. This evidence of the poor education, information and communication component of the LF MDA program in Bole is likely to have contributed to the non-compliance and persistent transmission of the disease in the district. This underscores the need for frontline staff (especially trusted healthcare workers) of the LF MDA program who educate the endemic community to have adequate training and the required knowledge about the disease and program. This evidence should motivate key educational messages that can be identified for pre-MDA stakeholder engagement, social mobilization and community sensitization campaigns to create awareness about the disease and the treatment.

### Challenges faced and suggestions to improve MDA

The challenges faced by CDDs and health workers include transportation-related difficulties, community accessibility, poor knowledge of the MDA among community members, CDD motivation and non-availability of community members and nonadherence to the DOT strategy. Similar factors were identified in other community health intervention in Ghana [11, 20, 21] and elsewhere [7, 8].

This 'study's 'participants' suggestion that there is the need to revisit households that were missed by the drug distributor during the first visit is consistent with the finding of another study in Ghana. The study suggested that drug distributors not revisiting homes where members were absent during the first visit contributes to affecting the drug intake in the community [21]. As the revisits will depend on the workload of the CDD, manpower and the time allotted for such purposes, this also requires incentivizing the health workers and the CDDs undertaking this task. Ultimately, it is in line with the fact that awareness must be created for the MDA and LF through better training of CDDs, massive education campaigns before the commencement of any drug distribution.

### Limitation

The combination of quantitative and qualitative methods employed in this study ensured a deeper understanding of the relevant factors affecting the quality of implementation of the LF MDA in the study. The results from each component of the study helped shed light, especially the qualitative study, which assisted in elucidating the findings of the quantitative study using key quotes. However, the following should be noted:

- The participants were interviewed a few months after the last MDA activities. Thus there is the chance of their inability to remember all the details of the activities before and during the previous MDAs. Therefore, recall bias cannot be entirely eliminated in this study.
- The findings may not be generalizable to all LF endemic districts given the peculiarities and specific contextual factors of the study districts.

Despite these limitations, this study makes valuable contributions to the existing literature on factors affecting the quality of implementation of LF MDAs in low resource settings.

### Conclusion and Recommendations

This study provides valuable insights that can be used to improve the quality of ongoing LF MDAs in hotspot districts to fast-track the interruption of the disease in those low-resource settings.

We observed a significant difference in the level of knowledge about the LF and the mass drug administration activities in the hotspot (Bole District) and stopped-MDA (Central Gonja District).

The persistent transmission of LF in Bole District is characterized by the refusal to ingest the drug, reported adverse drug reactions, low MDA coverage at the community level, and poor adherence to the MDA protocol.

High reported mass drug administration coverage (>65%) alone is not enough to interrupt transmission of LF. High MDA coverage coupled with strict adherence to the directly observe treatment strategy, strong stakeholder engagement, and evidence-based context-specific multi-channel community sensitization strategies with key educational messages on the cause of the disease and adverse drug reactions are necessary for the elimination of the disease.

Hence, while the clock is ticking for the elimination of LF by the year 2020 and meeting of the SDGs 3 target 3.3 by 2030, there is an urgent need for a concerted effort and focused attention to improve the fidelity of the ongoing LF MDA campaigns in the Bole District of Northern Ghana through strengthening the awareness and the involvement of all stakeholders.

We recommend the involvement of stakeholders such as NGOs, local leaders and self-help groups and use of evidence-based multi-channel community education strategies. The focus needs to be on key communication messages on adverse drug reactions and the creation of awareness on the causes of the disease. Additionally, vigorous enforcement of the DOT strategy during MDA training and supervision is recommended.

Due to the use of already collected data program data, we were not able to verify the accuracy of MDA coverages; hence we recommend coverage assessment surveys to validate reported MDA coverages.

## Supporting information

S1 Appendix Individual interview questionnaire. (DOCX)

S1 Table Summary statistics of MDA coverage in Bole and Central Gonja Districts. (DOCX)
