# Correction: Exploring factors affecting quality implementation of lymphatic filariasis mass drug administration in Bole and Central Gonja Districts in Northern Ghana

**DOI:** 10.1371/journal.pntd.0009341

**Published:** 2021-04-12

**Authors:** Alfred Kwesi Manyeh, Latifat Ibisomi, Rohit Ramaswamy, Frank Baiden, Tobias Chirwa

In the Knowledge on lymphatic filariasis subsection of the Results, there is an error in the second sentence of the first paragraph. The correct sentence is: The comprehensive knowledge of LF among the participants in Central Gonja District is higher compared to that of Bole District.
