# Supplementary material for: Exploring factors affecting quality implementation of lymphatic filariasis mass drug administration in Bole and Central Gonja Districts in Northern Ghana
Source: PLoS Negl Trop Dis. 2020 Aug 17;14(8):e0007009. doi: 10.1371/journal.pntd.0007009 (PMC7451553; doi:10.1371/journal.pntd.0007009)
Supplement: S1 Appendix — (DOCX) [file pntd.0007009.s001.docx]

**S1 Appendix: Individual interview questionnaire**

**UNIVERSITY OF THE WITWATERSRAND**

**FACULTY OF HEALTH SCIENCES, SCHOOL OF PUBLIC HEALTH**

TOWARDS ELIMINATION OF LYMPHATIC FILARIASIS IN GHANA: REFINING THE STRATEGY THROUGH QUALITY IMPROVEMENT.

| information panel | |
| --- | --- |
| Sub-districts: 1. Bole 2. Bamboi 3. Jama 4. Tinga 5. Mankuma 6. Mandari | |
| Community name: | Household code: |
| Name of Interviewer _________________________ | Date of interview: ___ /___ / __ |

*My name is ......................................................................................................................... and I am student of University of the Witwatersrand in Johannesburg, South Africa and want to ask some questions about the drug distribution for which the height of people were measured before the drugs were given. The purpose of this interview is to gather information that will help us to know what to do to reach all target population for effective elimination of LF in Ghana.*

**Seek consent**

**QUESTIONNAIRE**

| **Section A: Socio-Demographic Information** | | |
| --- | --- | --- |
| **No** | **Questions** | **Coding Categories** |
| 1 | **Age** | Age (Completed Years)……………… |
| 2 | **Sex** | Male………...………...............................................1  Female…………………………………….…............2 |
| 3 | **Marital Status** | Married…...…………………...................................1  Divorced…………………………….........................2  Separated………………………………….................3  Single……...…………………...................................4  Widowed………………………………….................5  Co-Habiting .............................................................6 |
| 4 | **Number of people in this household** |  |
| 5 | **Occupation** | Farmer…...…………...............................................1  Fisherman………………………..………….............2  Artisan………………………..…..…………............3  Trader…...………...................................................4  Public Servant………………………………............5  Unemployed…………………………….….............6  Other Specify…...…………....................................7 |
| 6 | **Religion** | Christian...................................................................1  Moslem....................................................................2  Traditional ……………............................................3  None........................................................................4  Other (specify)……………………….......................5 |
| 7 | **Highest level of education completed** | Primary....................................................................1  JHS/Middle/JSS........................................................2  Commercial/Vocational/Technical...........................3  A Level /SHS/Secondary..........................................4  Tertiary………………………………………............5  None........................................................................6  Other (specify).........................................................7 |

| **Section B: Knowledge about the Disease** | | |
| --- | --- | --- |
| 8 | **Have you heard about the disease Elephantiasis? (show a picture)** | Yes…………...…………….....................................1  No…….………………………………………........2 |
| 9 | How did you hear about the disease? | Disease control officer....................................,.........1  Community health nurses ........................................2  Public announcement ...............................................3  Media (TV, radio, newspapers, internet, etc.)...........4  Friend(s)....................................................................5  Relatives ...................................................................6  Community Volunteers…………………...……7  Other (specify) ..........................................................8  NA............................................................................88 |
| 10 | Do you know anyone with elephantiasis | Yes………...…………….........................................1  No.…………………….……………....……...........2  NA……………………………….……….............88 |
| 11 | **How is this disease acquired?** | Through the bite of a mosquito.....……..................1  Breathing in germs……………………...................2  Eating contaminated food........................................3  Drinking contaminated water……….……............4  Juju/Witchcraft/Spell...............................................5  Hereditary.................................................................6  Rain/ Stagnant water ..............................................7  Oily food .................................................................8  Poor hygiene ............................................................9  Other Specify..........................................................10  NA..........................................................................88  Don’t know.............................................................99 |
| 12 | **Which of the following would you associate with the disease?** | High body temperature (fever) ................................1  Swollen scrotum ......................................................2  Swollen breasts ........................................................3  Swollen legs .............................................................4  Contact with infected person ...................................5  Mosquitoes ...............................................................6  Sores on the body .....................................................7  Dirty environment ....................................................8  Others (specify) ........................................................9  NA............................................................................88  Don’t know ............................................................99 |
| 13 | Is there any treatment available to people with elephantiasis in this community? | Yes………...……….................................................1  No.………………….………………..……..............2  NA……………………………………….…..........88  Don’t know…………………………......................99 |
| 14 | What treatment does the health service have for disease? | Organize clean up campaigns……………….......... 1  Give drugs to the community………………...........2  Distribute bed nets…………………………............3  Treat those with the disease……….……................4  Other (specify)...........................…...........................5  NA…………………………………………..........88  Don’t know ............................................................99 |
| 15 | **How is elephantiasis disease prevented?** | By taking drugs........................................................1  Mass Drug Administration ......................................2  Sleeping in treated mosquito nets............................3  Keeping a clean environment...................................4  Insecticide spray/ Mosquito coil ..............................5  Other (specify)………...........................…...............6  NA..........................................................................88  Don’t know ............................................................99 |

| **Section C: Knowledge about the LF Mass Drug Administration** | | |
| --- | --- | --- |
| 16 | **Are you aware of any drug distribution in this community for which people’s heights were measured**? | Yes……..……............................................................1  No.……..........................………………..……….….2  NA..........................……...........…….……………..88  Don’t know……………………..........……………99 |
| 17 | **How is the community informed about this distribution program?** | Radio……………………………….........................1  Television……………………………......................2  Health workers……..……………….........................3  Posters……………………………….…...................4  Family members……….….…...................................5  Church/mosque………………….….........................6  Community volunteers…….……..............................7  Gong gong………………………..............................8  Neighbours/ friends……………................................9  Information van………….........................................10  Other, specify….…....................................................11  NA………………………………..….......................88 |
| 18 | **What is the purpose of the drug distribution exercise?** | To prevent/treat elephantiasis…….............................1  To prevent/treat hydrocele………………..................2  To prevent/treat onchocerciasis……..........................3  To prevent/ kill filaerial worms…………..................4  To reduce the incidence of LF………………...……5  Other specify……………………….….....................6  NA …………………………………........................88  Don’t know ...............................................................99 |
| 19 | **Was there public education before the distribution exercise?** | Yes…………...............................................................1  No.……………… ………..……..…..........................2  NA……………………….........................................88  Don’t know………….…………………..….............99 |
| 20 | **What would you want to know about the drug distribution?** | Drug safety and effectiveness……………...............1  Education about the disease………………..............2  Why the drug is not distributed in health facility .....3  Other specify…………………….…........................4  NA……………………………...............................88  Don’t know……………………..............................99 |
| 21 | **Where is the drug distribution done?** | Home to home ………………….……......................1  Community center ………………….......................2  At school…………………………….……...............3  At marketplace……………………….…….............4  Chief’s palace …………………………...................5  At church…………………………….…..................6  Other (specify)…… ……………………..................7  NA...........................................................................88  Don’t know ............................................................99 |
| 22 | **How do they (Volunteers) do the drug distribution?** | Measure height before drugs are given……..............1  Ask about health status...……………………..........2  Ask about alcohol intake ……………….…........... .3  Ask about age…………………………................…4  Directly observe treatment……………................…5  Ask whether one has eaten………………...........….6  Explain the reasons for the treatment...................….7  Other specify …………………………...............…..8  NA ……………………………………..............…88  Don’t know …………………………....................99 |
| 23 | **What categories of people are not given the drugs?** | Children less than a certain height/age......................1  Pregnant women........................................................2  Breast feeding mothers .............................................3  Sick people................................................................4  If you have not eaten…………..………...................5  Others specify… .......................................................6  NA...........................................................................88  Don’t know ............................................................99 |
| 24 | **How was the drug delivered?** | Direct observation treatment......................................1  Given to the beneficiary to take at their convenient...2  Other (specify)……………………………................3 |
| 25 | **What reasons do people give for refusing to take the drugs?** | Fear of side effects………………………...............1  Lack of education on the disease………….............2  Don’t think will get the disease……………...........3  Think only sick people should take…….................4  Too many drugs…………………………...............5  Religious beliefs/superstition.…………….............6  Taking other medication………………..................7  Have taken the drugs for too many times............…8  Other (specify)………..…………….......................9  NA……………………………………...................88  Don’t know .............................................................99 |
| 26 | **What are some of the side effects of taking these drugs?** | Itching........................................................................1  Rashes........................................................................2  Swelling of parts of the body.....................................3  Headache...................................................................4  Fever..........................................................................5  Chills.........................................................................6  General muscle/bodily pains.....................................7  Fainting......................................................................8  Vomiting....................................................................9  Diarrhoea……………………………….................10  Other specify……………………............................11  NA……………………………………....................88 |
| 27 | **Do you consider the treatment important?** | Yes ............................................................................1  No .............................................................................2  Don’t know ..............................................................99 |
| 28 | **What suggestions do you have for improving future mass drug distribution exercises?** | Create awareness…….…………..……....................1  Health workers should distribute…..….....................2  Volunteers should come from the community…..…3  Distribute during the dry season……...................….4  Distribute during the rainy season…….....................5  Extend the distribution period………..................….6  Provide food with the drugs…………..................…7  Distribute at work places………………...................8  Distribute at hospitals…………………....................9  Train volunteers well…………….……..................10  Distribute the drugs at vantage points….................11  Distribute on weekends/ morning/ evening ……....12  Distribute in churches/mosques….…......................13  Other, specify……...........................................…...14  Don’t know .............................................................99 |
