# Supplementary material for: Exploring factors affecting quality implementation of lymphatic filariasis mass drug administration in Bole and Central Gonja Districts in Northern Ghana
Source: PLoS Negl Trop Dis. 2020 Aug 17;14(8):e0007009. doi: 10.1371/journal.pntd.0007009 (PMC7451553; doi:10.1371/journal.pntd.0007009)
Supplement: S1 Table — (DOCX) [file pntd.0007009.s002.docx]

**S2 Table: Summary statistics of MDA coverage in Bole and Central Gonja Districts**

| **Summary Statistics** | **Central Gonja District** | | **Bole District** | |
| --- | --- | --- | --- | --- |
|  | **Community Coverage** | **Sub-district Coverage** | **Community Coverage** | **Sub-district Coverage** |
| Mean | 87.7 | 89.3 | 82.7 | 81.3 |
| Standard Deviation | 6.8 | 0.7 | 12.2 | 14.0 |
| Range | 70.9 - 100 | 88.4 – 90.2 | 47.5 - 100 | 70.0 – 98.9 |
